# Supplementary material for: Antibacterial Activity and Mode of Action of Lactoquinomycin A from Streptomyces bacillaris
Source: Mar Drugs. 2020 Dec 24;19(1):7. doi: 10.3390/md19010007 (PMC7823745; doi:10.3390/md19010007)
Supplement: Supplementary file 1 [file marinedrugs-19-00007-s001.pdf]

Supporting Information

Antibacterial Activity and Mode of Action of Lactoquinomycin A from *Streptomyces bacillaris*

Beomkoo Chung [1], Oh-Seok Kwon [2], Jongheon Shin [2,*], and Ki-Bong Oh [1,*]

[1] Department of Agricultural Biotechnology, College of Agriculture and Life Sciences, Seoul National University, Seoul 08826, Korea; beomkoo01@snu.ac.kr
[2] Natural Products Research Institute, College of Pharmacy, Seoul National University, Seoul 08826, Korea; ideally225@snu.ac.kr
* Correspondence: shinj@snu.ac.kr (J.S.); ohkibong@snu.ac.kr (K.-B.O.); Tel.: +82-2-880-2484 (J.S.); +82-2-880-4646 (K.-B.O.)

Contents

Table S1. ^{13}C NMR compare **1** with Lactoquinomycin A in $CDCl_3$.

Table S2. ^{13}C NMR compare **2** with Lactoquinomycin B in MeOH-d_4, $CDCl_3$, respectively.

Table S3. ^{13}C NMR compare **3** with N-methyl lactoquinomycin A in MeOH-d_4.

Table S4. ^{13}C NMR compare **4** with Menoxymycin A in MeOH-d_4.

Figure S1. ^{1}H NMR of compound **1** in $CDCl_3$

Figure S2. ^{13}C NMR of compound **1** in $CDCl_3$

Figure S3. ^{1}H NMR of compound **2** in MeOH-d_4

Figure S4. ^{13}C NMR of compound **2** in MeOH-d_4

Figure S5. ^{1}H NMR of compound **3** in MeOH-d_4

Figure S6. ^{13}C NMR of compound **3** in MeOH-d_4

Figure S7. ^{1}H NMR of compound **4** in $CDCl_3$

Figure S8. ^{13}C NMR of compound **4** in $CDCl_3$

Table S1. ^{13}C NMR compare **1** with lactoquinomycin A in $CDCl_3$.

	**1**	**Lactoquinomycin A**
	δ_C, ppm	
1	66.2	66.3
3	66.4	66.5
4	68.5	68.7
4a	135.5	134.9
5	181.1	180.8
5a	130.5	129.7
6	119.9	119.6
7	133.8	133.5
8	136.5	138.6
9	157.7	157.7
9a	114.3	114.0
10	188.5	187.8
10a	149.6	149.2
11	36.9	37.0
12	173.9	173.5
1-CH_3	18.1	18.8
1'	71.1	72.2
2'	29.3	28.2
3'	68.0	67.2
4'	70.4	71.5
5'	77.7	77.6
3'-$N(CH_3)_2$	40.1	40.3
6'-CH_3	18.6	18.9

Table S2. ^{13}C NMR compare **2** with lactoquinomycin B in MeOH-d_4, $CDCl_3$, respectively.

	**2**	**Lactoquinomycin B**
	δ_C, ppm	
1	64.3	64.2
3	65.0	64.8
4	67.1	69.2
4a	60.5	60.0
5	187.3	187.6
5a	130.8	129.7
6	119.1	120.0
7	133.3	134.2
8	136.6	139.1
9	157.6	157.8
9a	114.0	113.5
10	195.2	193.9
10a	64.4	64.4
11	36.0	35.6
12	175.8	173.3
1-CH_3	13.3	15.2
1'	71.0	72.4
2'	28.9	28.1
3'	69.8	67.2
4'	70.3	71.5
5'	77.2	77.6
3'-$N(CH_3)_2$	40.7	40.3
6'-CH_3	16.8	19.0

Table S3. ^{13}C NMR compare **3** with N-methyl lactoquinomycin A in MeOH-d_4.

	**3**	**N-methyl lactoquinomycin A**
	δ_C, ppm	
1	66.4	67.7
3	66.8	68.1
4	69.5	70.8
4a	135.4	136.7
5	181.5	182.8
5a	130.8	132.2
6	118.7	120.0
7	133.2	134.4
8	136.5	137.9
9	157.5	158.8
9a	114.6	116.0
10	188.7	190.1
10a	149.6	151.0
11	36.3	37.7
12	175.9	177.3
1-CH_3	17.2	18.5
1'	71.0	72.3
2'	32.6	34.0
3'	60.7	62.0
4'	72.2	73.6
5'	77.1	78.4

3'-N-CH_3	29.2	30.6
6'-CH_3	16.9	18.2

Table S4. ^{13}C NMR compare **4** with menoxymycin A in MeOH-d_4.

	**4**	**Menoxymycin A**
	δ_C, ppm	
1	66.2	66.2
3	68.6	68.5
4	66.5	66.4
4a	136.3	135.4
5	181.1	181.1
5a	130.6	130.4
6	119.8	119.8
7	133.8	133.8
8	135.5	136.9
9	157.7	157.8
9a	114.3	114.3
10	188.4	188.4
10a	149.6	149.6
11	36.9	36.9
12	174.0	173.9
1-CH_3	17.9	17.8
1'	71.2	72.9
2'	32.2	29.7
3'	77.3	75.9
4'	71.0	71.3
5'	78.5	77.8
3'-NO$(CH_3)_2$	56.6, 55.5	58.4, 52.7
6'-CH_3	18.5	18.5

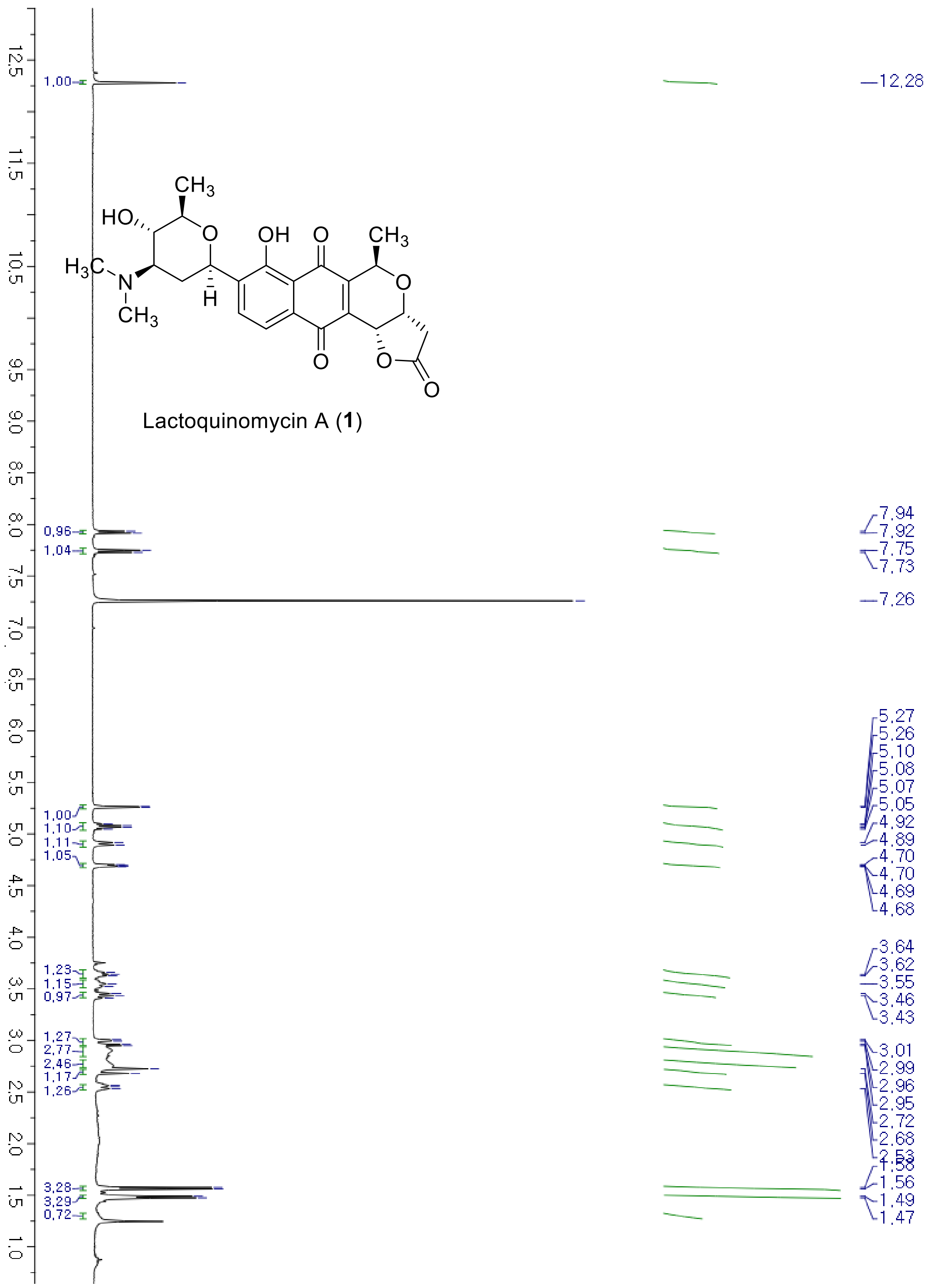

Figure S1. ^{1}H NMR of compound **1** in $CDCl_3$.

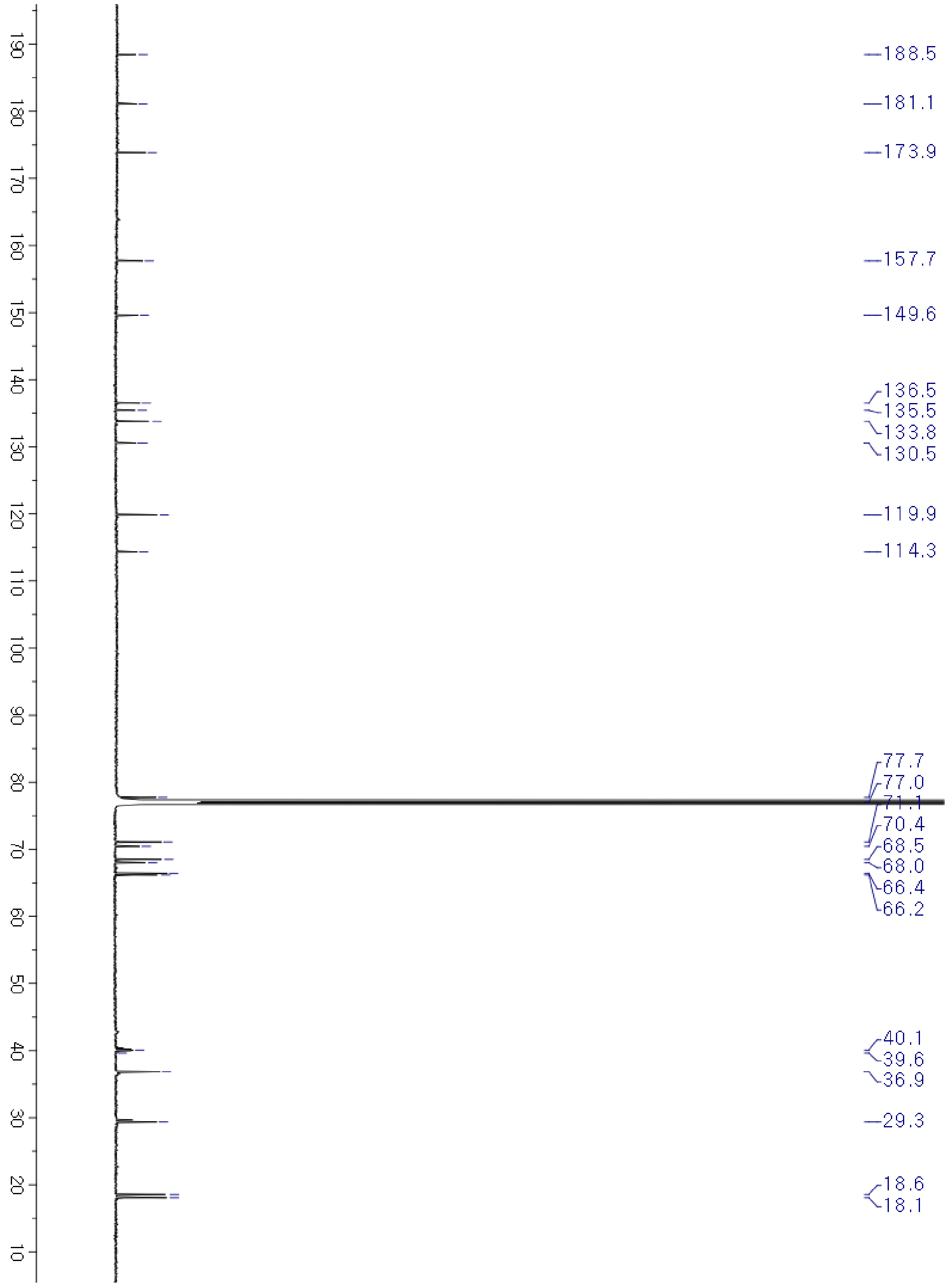

Figure S2. ^{13}C NMR of compound **1** in $CDCl_3$.

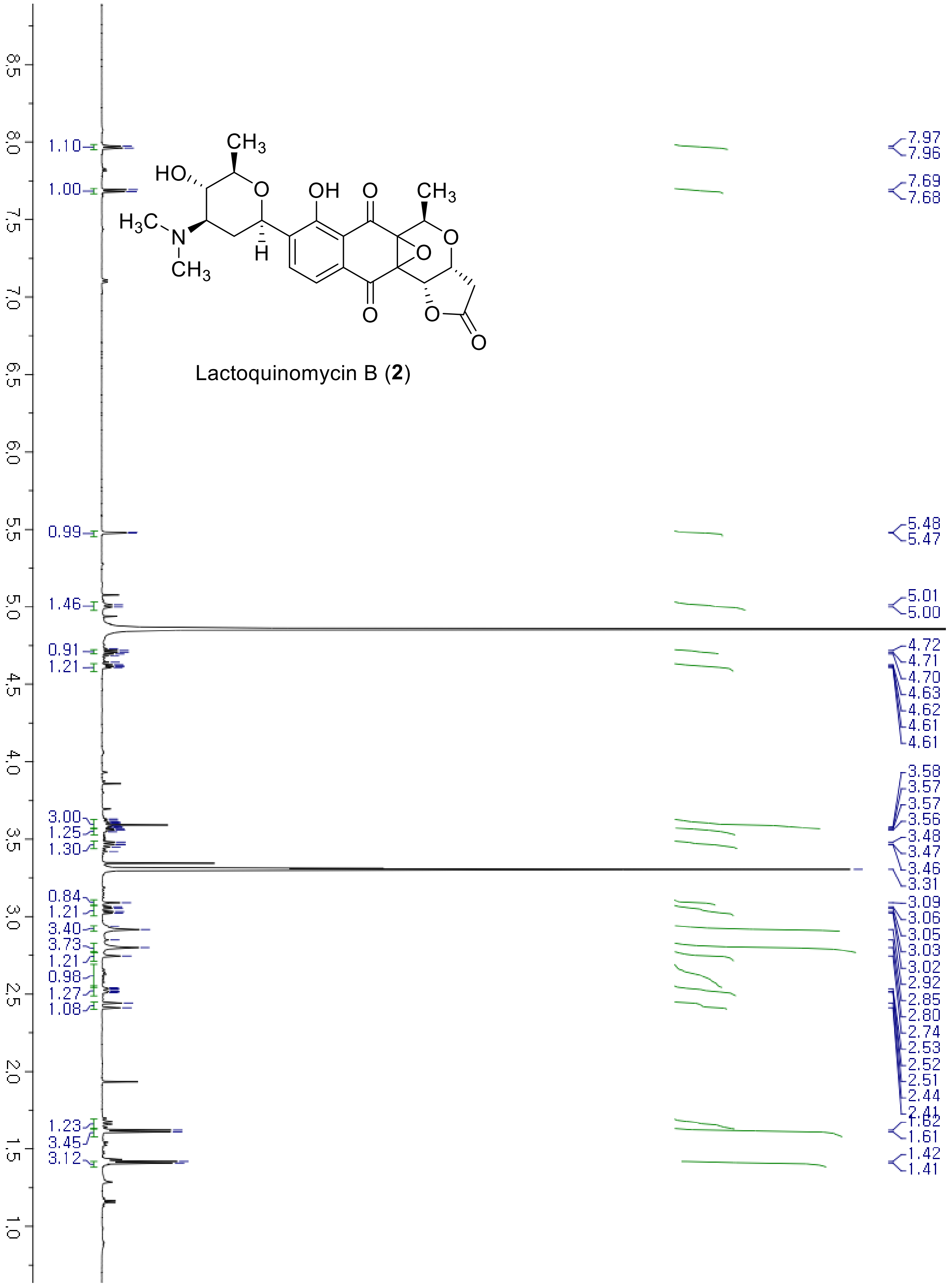

Figure S3. ^{1}H NMR of compound **2** in MeOH-d_4.

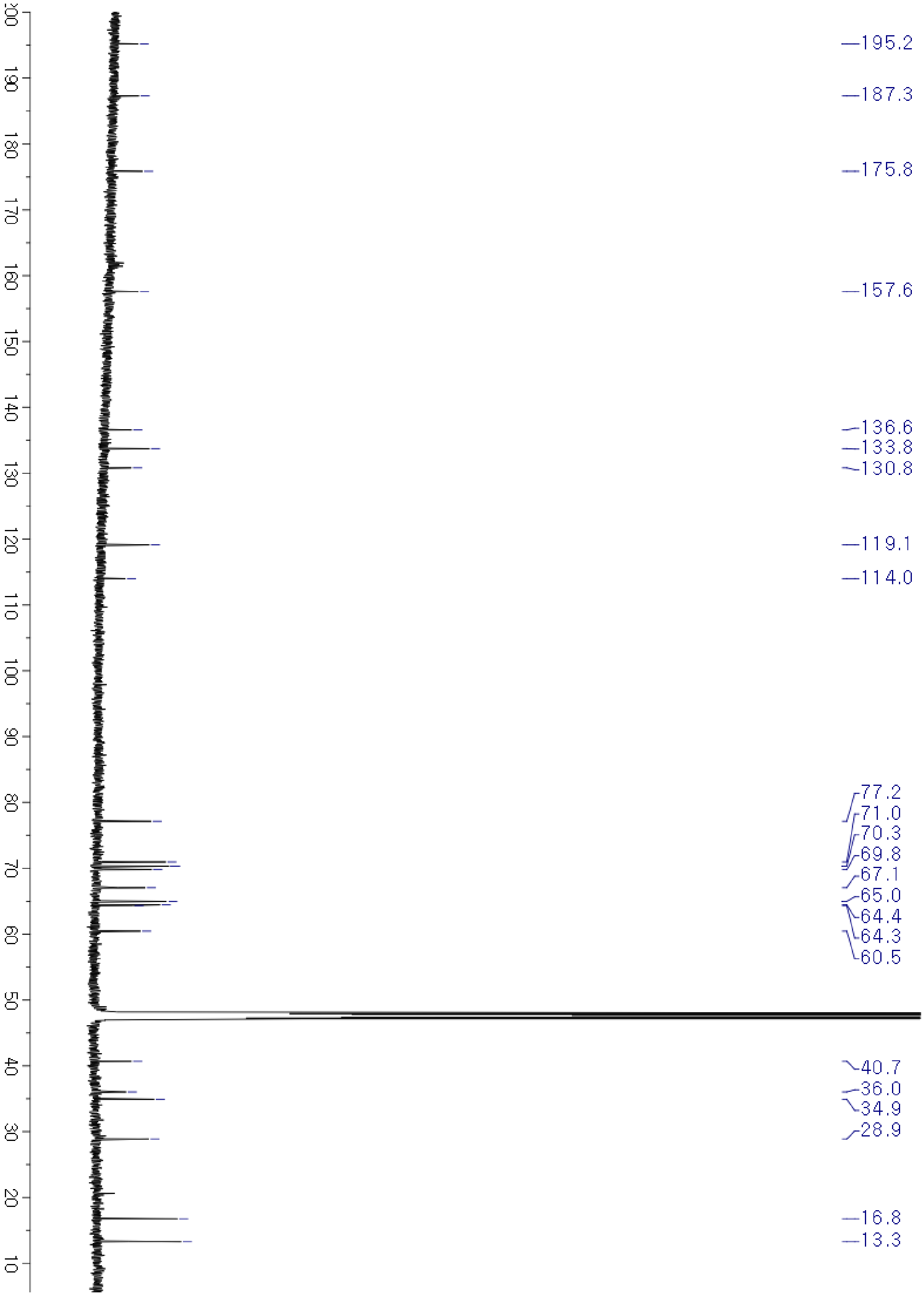

Figure S4. ^{13}C NMR of compound **2** in MeOH-d_4.

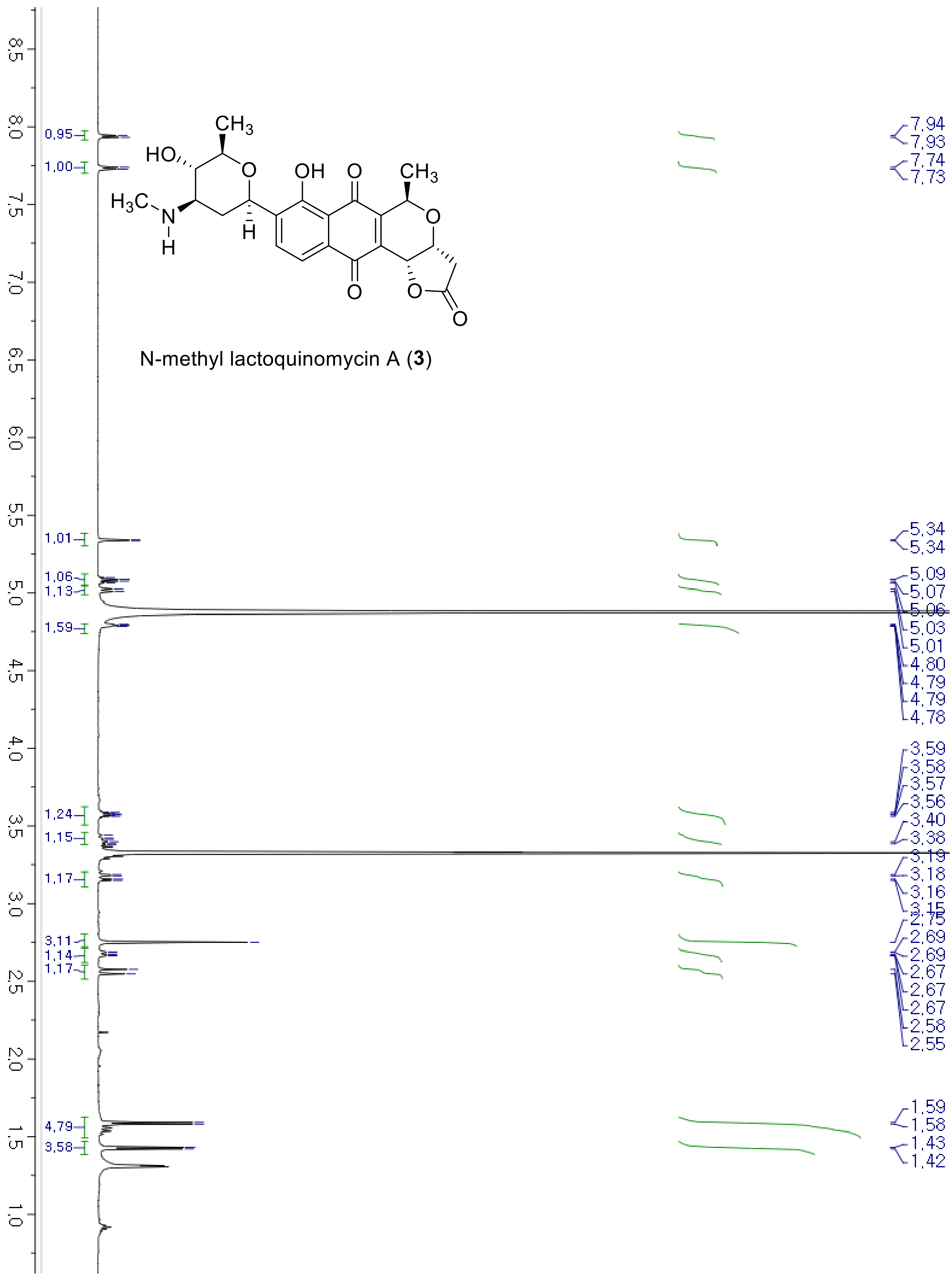

Figure S5. ^1H NMR of compound **3** in MeOH-d_4.

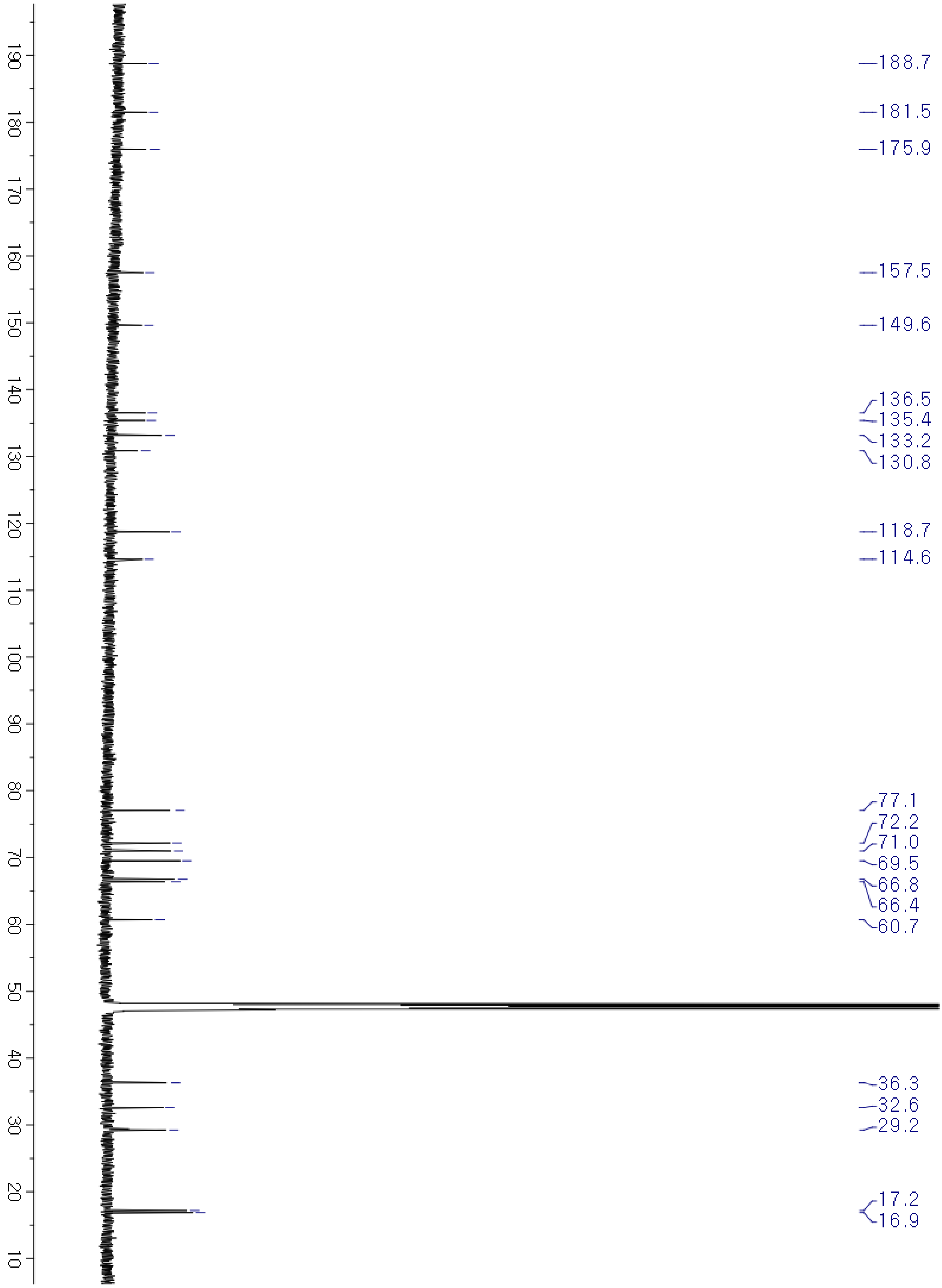

Figure S6. ^{13}C NMR of compound **3** in MeOH-d_4.

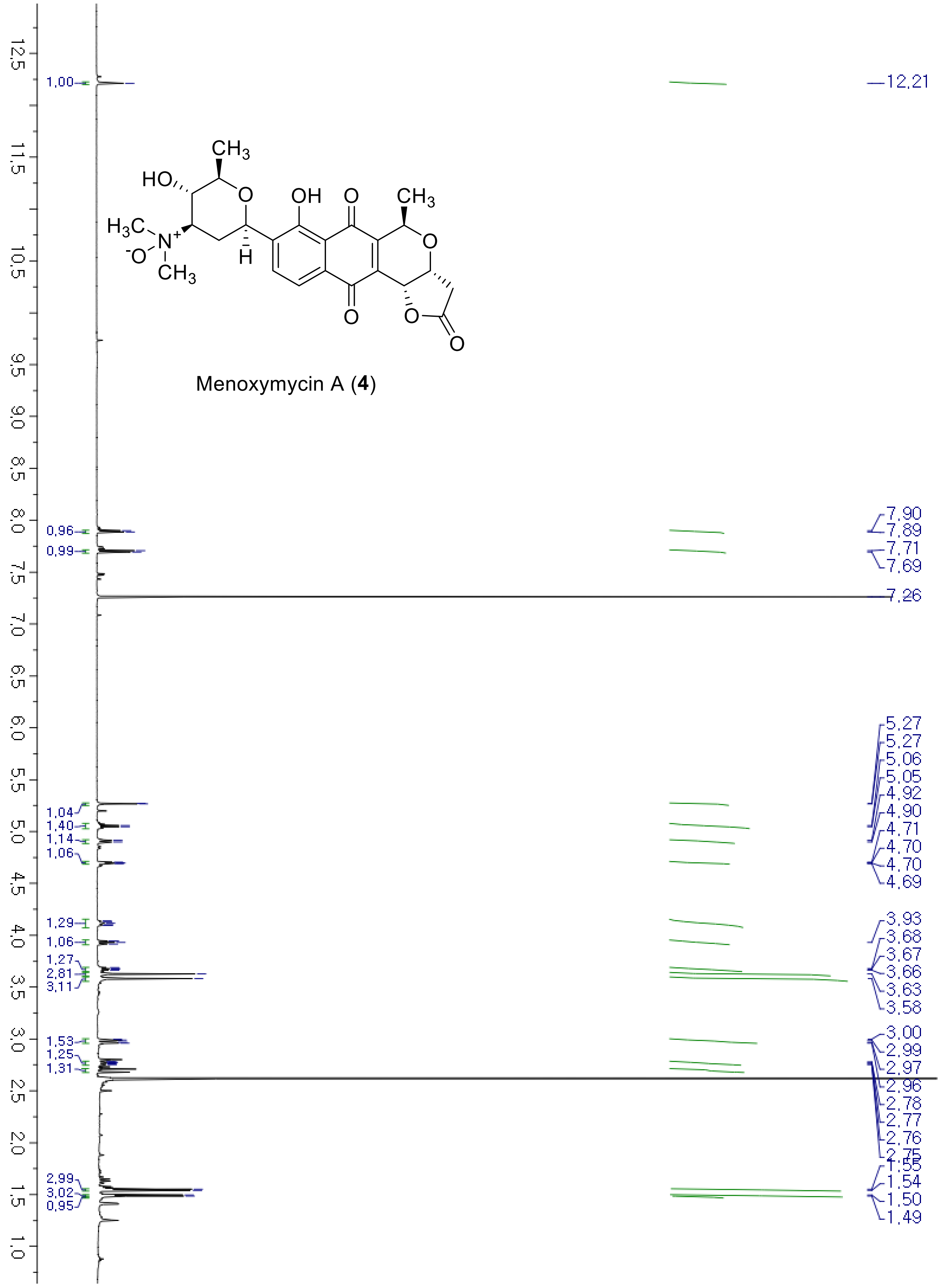

Figure S7. ^{1}H NMR of compound **4** in $CDCl_3$.

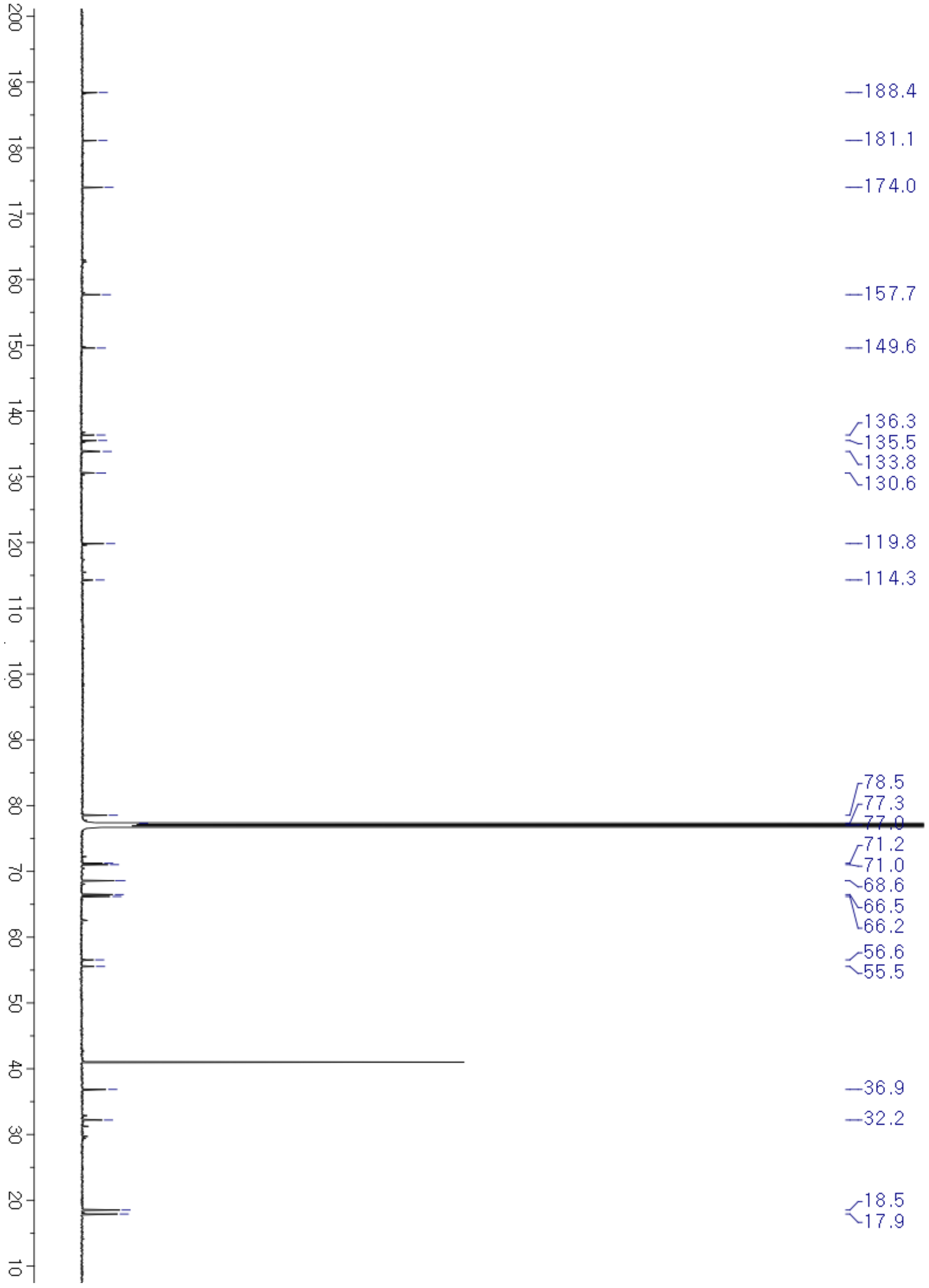

Figure S8. ^{13}C NMR of compound **4** in $CDCl_3$.
